# Presenting Evidence to Patients Online:  What Do Web Users Think of Consumer Summaries of Cochrane Musculoskeletal Reviews?

**DOI:** 10.2196/jmir.1532

**Published:** 2011-01-18

**Authors:** Jamie C Brehaut, Nancy Santesso, Annette M O'Connor, Alison Lott, Gitte Lindgaard, Ania Syrowatka, Ian D Graham, Peter S Tugwell

**Affiliations:** ^8^Institute of Population HealthUniversity of OttawaOttawa, ONCanada; ^7^Knowledge Translation PortfolioCanadian Institutes of Health ResearchOttawa, ONCanada; ^6^Human Oriented Technology LaboratoryDepartment of PsychologyCarleton UniversityOttawa, ONCanada; ^5^Knowledge Synthesis and Exchange BranchCanadian Institutes of Health ResearchOttawa, ONCanada; ^4^School of NursingUniversity of OttawaOttawa, ONCanada; ^3^Clinical Epidemiology ProgramMcMaster UniversityHamilton, ONCanada; ^2^Epidemiology and Community MedicineUniversity of OttawaOttawa, ONCanada; ^1^Clinical Epidemiology ProgramOttawa Hospital Research InstituteThe Ottawa HospitalOttawa, ONCanada

**Keywords:** Decision support techniques, health care surveys, Internet, patient education, patient satisfaction, patient-centered care, musculoskeletal diseases

## Abstract

**Background:**

The Internet has the potential to be an effective medium for delivering health care knowledge to consumers. While computer usability research makes recommendations about how to present Web-based information generally, there remains no clear guidance on how to present specific forms of health care research evidence online in a way that facilitates understanding and good health care decision making.

**Objective:**

The two goals of this study were to describe the Cochrane Musculoskeletal Group’s (CMSG’s) process for developing online patient-focused summaries of systematic reviews and to evaluate the impressions of these summaries formed by users.

**Methods:**

A process for summarizing the results of systematic reviews via consumer summaries has evolved over 15 years. An evaluation of this approach took the form of Internet surveys on the Arthritis Society of Canada website and surveys of members of the Canadian Arthritis Patient Alliance (CAPA). Respondents provided information on background, relationship to the decision, their satisfaction with and preparation for decision making, and suggestions for improvements to the summaries. Survey data were collected between August 1, 2005, and February 28, 2006.

**Results:**

A total of 261 respondents completed the survey. The majority (226/261 or 87%) of respondents reported having an arthritis-related condition. The consumer summary approach was generally reviewed favorably by respondents, with most agreeing that the summary provided appropriate information (177/261 or 68%), would be useful to others (160/261 or 61%), was well laid out (159/261 or 61%), was easy to learn from (157/261 or 60%), and was useful to the reader (153/261 or 59%). Areas of potential improvement were indicated by relatively fewer respondents agreeing that they could easily find all the information they wanted (118/261 or 45%), by a substantial proportion being unable to judge whether the providers of the information are reliable (80/261 or 31%), and by a similar proportion being unable to determine whether the information presented was the best available (68/261 or 26%).

**Conclusions:**

The CMSG has developed an approach to summarizing the results of often-technical systematic reviews into public-friendly consumer summaries. Our online survey showed that this approach was generally well liked but identified specific areas for improvement. Feedback from this survey will help to reshape and improve the current template for consumer summaries used by the CMSG.

## Introduction

### Background

A key aspect of the rapidly changing face of health care is the explosion of knowledge targeted at health care consumers. In part, because of advancing information technology allowing access to them, knowledge producers are increasingly seeing consumers as an important target group. In a recent review of 56 Canadian organizations producing practice guidelines between 2000 and 2005, it was found that 630 unique guidelines had been developed; of these, 42.7% included consumer versions or were intended for consumers [1]. Furthermore, many major governmental and nonprofit organizations such as the UK National Institute for Health and Clinical Excellence (NICE) [2], the US Agency for Healthcare Research and Quality (AHRQ) [3], the Journal of the American Medical Association [4], the Cochrane Collaboration [5], along with many patient condition-specific organizations (eg, Arthritis Society of Canada [6]) now disseminate research evidence directly to health care consumers, primarily via the Internet. Indeed, targeting patients can be an effective strategy to reduce the gap between research knowledge and clinical practice [7,8].

Development of consumer-targeted health knowledge is not only on the rise because of “push” from knowledge producers but also because of “pull” by consumers [9]. Consumers are often highly motivated to maximize the quality of their own care and are demanding greater involvement in decisions surrounding their own health care [10]. This is manifested as a demand for health care knowledge, with the Internet serving as an important mode of delivery. For example, about 80% of Canadians over the age of 16 now report using the Internet, with health information being the most commonly reported search topic [11,12].

The Internet has many advantages as a medium for delivering health care knowledge to consumers. For knowledge producers, the Web can provide wide distribution at relatively little cost. Its potential for interactivity can allow both an improved learning environment and data gathering alongside dissemination activities. Furthermore, knowledge can be updated with relative ease and low cost when compared to print or other media. For consumers, the Web is accessible, free, convenient, and allows for learning at a pace the individual finds most comfortable.

Despite these advantages, effective dissemination of online health care knowledge can be limited by variable quality and indeterminate reliability [13]. Relevant literatures exist, but have not been exploited. Computer usability research makes general recommendations about presenting Web-based information [14,15], but these lessons have often not been followed in presenting health information online. Similarly, considerable research from the patient decision-support and human decision-making literatures [16,17] has not been translated into specific recommendations for facilitating good decision making based on online health information. We propose that specific approaches to presenting health care research knowledge online to consumers need to be developed and evaluated.

The Cochrane Musculoskeletal Group (CMSG) has produced consumer summaries of over 100 of its systematic reviews of health care interventions for arthritis and other related conditions. These consumer summaries are targeted at patients but have only recently become widely available on the Internet and are now accessible by over half of the world’s population through country-level subscriptions to the Cochrane Library and consumer-targeted websites (eg, Arthritis Society of Canada [6] and Arthritis Victoria in Australia [18]).

While these summaries were developed with extensive consumer input, they were primarily developed in a paper format, which may not have translated well into effective online information tools. Guidance for producing effective online tools comes from at least two sources. First, the human-computer interaction literature identifies components of effective online information, as well as means to measure them [15]. For example, the extent to which a computer website is favorably rated by users has been categorized into 5 domains of satisfaction including aesthetics, likeability, usability, emotion, and expectation [14,19]. Second, a substantial literature has grown up around the Ottawa Decision Support Framework [16,20,21] focused on measuring constructs related to good-quality decision making. Based on this literature, we sought to examine the extent to which the CMSG summaries were seen to be relevant to such constructs.

### Objectives

This paper had two primary objectives: (1) to provide a narrative describing the work of the CMSG in creating online consumer summaries of the evidence from systematic reviews and (2) to evaluate the impressions of these summaries formed by users of the Arthritis Society of Canada website via an Internet survey. This knowledge will contribute toward establishing guidelines about how to summarize and present research evidence to consumers on the Internet.

### The Cochrane Musculoskeletal Group Consumer Summaries

The CMSG consumer summaries have evolved over the last 15 years. Initially developed on an ad hoc basis, they have been revised and standardized based on the recommendations of a variety of guidelines for creating patient information [22-24], evidence from research [25], and user feedback. Summaries are regularly distributed and feedback sought from consumer members of the CMSG at annual CMSG meetings and from consumers and research professionals at workshops at Cochrane Colloquium meetings [26,27].

**Figure 1:**
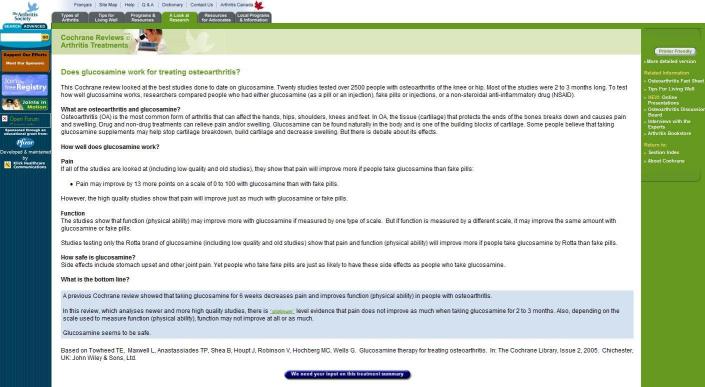
Example of a consumer summary

What has resulted is the standard 1-page summary now used by the CMSG (see example, Figure 1). Each summary usually consists of no more than 400 words and typically takes consumers approximately 5 minutes to read. Each summary is divided into short sections with illustrative, standardized questions as headers. An introductory section (section 1) provides background on what and who was studied and mentions the Cochrane Collaboration as the source of the information. Section 2 answers questions about the intervention and the condition, for example, “What are osteoarthritis and glucosamine?” Section 3 answers questions about the effectiveness of the intervention, for example, “How well does glucosamine work?” Section 4 answers questions about safety, explicitly addressing both benefits and harms, for example, “How safe is glucosamine?” The final section provides a single-statement summary of the overall meaning of the results and provides a Web link to the description of the level of evidence underlying these statements (ie, platinum, gold, silver, and bronze levels of evidence). This method for grading scientific evidence was derived by Tugwell et al and incorporates the types of studies and quality of evidence into the ranking [28]. This section answers the question, “What is the bottom line?”

Sections are kept short, and information is provided in a logical flow to facilitate interpretation by a wide range of audiences. While formal readability evaluations have been carried out at various points during the development process of these summaries, questions about the validity of formal readability evaluations have been noted [29]. We found that for these summaries the technical terminology describing the diseases and treatments in CMSG reviews often inflated readability scores, while eliminating this terminology to reduce readability scores often caused more problems than it solved for users. Rather than relying on formal readability scores, we opted to ensure that all technical terms were clearly defined, and that all language was clear and readable. We saw readability analysis, therefore, as a means to end (a readable document dealing with technical issues) rather than an end itself (a document with a particular readability score).

We also strove to maintain the active voice throughout, based on the recommendations of a number of guidelines [30-32]*.* When available, outcome probabilities are presented in natural frequencies with consistent denominators (eg, “72 out of 100”), explicitly describe the time frame to which the results apply (eg, “after 6 months of treatment, 10 patients out of 100 will improve will improve”), and present the numbers in multiple ways (eg, “this means that 12 more people will improve”) to facilitate understanding [16]. Table 1 describes what we see as key components of successful summaries as informed by the experience of the CMSG development process. While these individual recommendations have not been empirically tested in the current context, their combination gave rise to the summaries evaluated here.

**Table 1:** Preliminary recommendations for presenting consumer summaries online

Recommendations	
1	Consider existing standards for clear presentation of risk information (eg, [16])
2	Pilot test individual summaries on the target audience
3	Provide links to definitions for technical terms
4	Maintain consistent formatting between summaries
5	Consider providing information-rich displays (eg, charts and graphs) in addition to clear language
6	Consider the readability, or in cases where technical terms are unavoidable, the lexical density of the language
7	Indicate level of evidence supporting risk estimates (eg, gold and silver)
8	Provide links to more detailed information
9	Prominently display information on the provider and timeliness of the information
10	Keep summaries short (at approximately 400 words) and provide short bottom-line statements of key messages

In total, over 100 consumer summaries of systematic reviews for treatments of arthritis-related conditions have been produced and made available by the CMSG between 1993 and 2005. All are available online through the Arthritis Society of Canada website [6] and were the focus of our online survey. This site contains a wide variety of information about the many conditions related to arthritis, tips for living well, drug information, discussion forums, self-management programs, and research information. The consumer summaries are located in the latter section of the website and can be reached in 2 clicks from the home page. The summaries are organized by type of arthritis, and links are provided to related information on the website. To evaluate and improve the summaries, we conducted a Web-based survey of a subset of visitors to the Arthritis Society Website who read 1 or more CMSG summaries.

## Methods

### Survey: Overall Approach

Over the years, CMSG summaries have been subjected to many rounds of focus groups, interviews, and other forms of qualitative testing and evaluation. We chose to conduct an evaluation using a Web-based survey for 2 main reasons. First, the majority of this work was carried out on paper-based summaries, and we wanted to know how well the summaries translated to an online environment. Second, most of this qualitative testing was carried out on a select sample of people who were closely aligned with the CMSG, and we wanted to elicit the impressions of a wider range of types of respondents. In addition, while our survey should not be considered theory-derived, the choice of constructs was primarily informed by principles of computer usability [15,19] and the Ottawa Decision Support Framework [20,21], around which constructs relevant to good quality decision making have been developed.

### Survey Development

This study was approved by the Ottawa Hospital Research Ethics Board. We designed an exploratory Web-based survey to evaluate user impressions of the CMSG consumer summaries. After the author group identified all the key constructs to be included in the survey, reviews of the literature were carried out for validated measures of the relevant constructs, and, where appropriate, such measures were included in the original survey or versions modified for Web-based administration. An iterative process of design, evaluation by the author team, and redesign resulted in a draft version of the survey that was programmed for the Web. The survey was then pilot tested at the Carleton University Human Oriented Technology Laboratory by 5 senior students trained in issues of computer usability. These students completed the survey and provided feedback on how to improve the aesthetic qualities, layout, content, and navigational ease of the survey.

The final version of the survey consisted of a cover letter and 5 sections and included both open-ended questions and closed-ended questions with pull-down menu or check box-type response options.

The cover letter of the survey included the names of principal investigators on the project, the purpose of the study, and the length of time the survey was expected to take. It also stated that completion and submission of the survey would serve as tacit consent that the subject’s responses could be used in the study. Details of duration and location of data storage were not provided unless specifically requested by the participant.

The survey consisted of 5 sections. The first addressed summary-specific issues such as how long participants had spent reading the summary. Subsequent sections included items on user experience, satisfaction with the way the information prepared them for decision making, experience with computers, background/demographic information, and suggestions for improvement. The complete survey consisted of 1 screen displaying a total of 53 items.

### Survey Questions

Summary-specific items measured the time spent reading the summary as an indirect measure of the care with which reviewers read the items (categorized in 5-minute increments). Also measured was confidence in participants’ understanding of the key points of the summary (on a 5-point scale from “not at all” to “very” confident) as a key prerequisite of good decision making as described by the Ottawa Decision Support Framework [20,21]. Finally, items targeting the extent to which the respondent was closely related to the issue included how participants had discovered the treatment summaries (ie, by exploring arthritis.ca, through a search engine, a recommendation of a physician, friends/family, or a support group, or other); participants’ main reasons for reading the summary (ie, for personal relevance, general interest, or other), and interest in arthritis (ie, “I have arthritis,” “I know someone with arthritis,” or other). The summary the respondent had read was logged automatically.

Computer user experience was measured using 14 items (Table 4) related to 5 domains of user satisfaction [19] including (1) aesthetics, (2) likeability, (3) usability, (4) emotion, and (5) expectation. Each item was rated on a 6-point scale from “strongly disagree” to “strongly agree” with an option of “no opinion/don’t know.” Also, 4 items (the summary is boring, the summary is frustrating, learning from the summary seems hard, I had to read too much) were phrased in the opposite direction from the rest of the items, and reported item means were reversed for ease of comparison.

Satisfaction in how the information materials prepared them for decision making was measured using 11 items adapted from Graham and O’Connor [33]. Items elicited respondents’ feelings of support and preparation to make decisions (Table 5). Items were rated on a 5-point scale from “not at all” to “a great deal.” Finally, an open-ended question asked for advice on how the consumer summaries could be improved. Note that items from both the user experience and satisfaction with preparation for decision-making instruments were analyzed separately, as neither scale had been validated in the form administered in this survey. Our goal for these items was to describe people’s impressions of the consumer summaries rather than target potential underlying constructs. Analysis and validation of underlying constructs for these items will be the subject of separate investigation.

We included 6 computer expertise items selected from a scale reported by Liaw et al [34], which asked respondents to self-report on their experience with (1) computers in general, (2) the Internet/World Wide Web, (3) Internet search engines, (4) word processing software, (5) database software, and (6) computer programming languages. Participants were asked to rate each of these 6 items on a 5-point scale ranging from 0, “none at all” to 4, “a great deal.” The 6 items were summed to produce a scale score ranging from 0 to 24. We then categorized this scale into thirds to indicate respondents with low, middle, and high levels of self-reported computer experience.

Background questions included demographic questions about sex, age, education, location of residence, and employment status.

### Sampling and Recruitment

Sample size for this descriptive survey was based on estimates of the amount of traffic to the website and the response rate to the online questionnaire. We chose to aim for 300 respondents to yield a wide range of opinions on each of the 10 summaries attached to the survey. In prior years, approximately 17,000 users visited the relevant section of the Arthritis Society of Canada website. From this pool, previous (1-question) surveys administered on this website had typically yielded 200 to 500 responses per month. Based on an estimated completion rate of 30% of that number, we planned to collect 60 to 150 responses per month and take between 2 and 5 months to collect the data.

Recruitment to the survey proved a bigger challenge than expected. As a result, we engaged in 3 relatively separate recruitment strategies. First, we used a convenience sampling strategy in which the administrator of the Arthritis Society of Canada website posted a link on the home page of the site encouraging any visitors to the site to consider completing a voluntary, Web-based, open version of the survey. Respondents were assured of the confidentiality of their data, but this version did not specifically state that ethics board approval had been granted. Second, the society distributed a letter on behalf of the study authors to the provincial Arthritis Society educational team leaders asking them to encourage their local members and contacts to complete the Web-based survey. Finally, an electronic invitation to participate in a closed version of the survey was sent to all 463 members of the Canadian Arthritis Patient Alliance (CAPA), an advocacy group closely related to the Arthritis Society of Canada. Members of CAPA typically have arthritis or have a strong interest in arthritis advocacy.

### Administration

The Web survey was linked to 10 of the most popular CMSG summaries on the Arthritis Society of Canada website. All 10 summaries were standardized with regard to format and content as of July 2005 (see Figure 1).

Respondents chose the summary that was most relevant to them either by clicking on it during their Internet session or by being directed by an email recruitment letter to a list of the 10 target summaries. A link to the survey itself was appended to the bottom of each selected summary, and instructions on the survey indicated that participants should complete the survey about the summary they had just read. Summary topics addressed issues around various treatments for osteoarthritis, rheumatoid arthritis, and shoulder and elbow pain. Each summary generally described the effects of a single intervention, such as drug interventions (eg, methotrexate or glucosamine) or nonpharmacologic treatments (eg, exercise or ultrasound).

To the extent possible, administration of the closed version of the survey to CAPA members was governed by Dillman’s Tailored Design Method [35]. A prenotification to complete the survey, an invitation to visit the summaries and complete the survey, and 2 reminder emails were sent via email. This version of the survey consisted of 1 screen displaying a total of 71 items including most questions from the earlier version (with the exception of 2 arthritis.ca website feedback items) plus 1 additional scale, the Medical Data Interpretation Test [36], which was excluded from analysis due to technical problems. An appendix with the CAPA prenotification, CAPA cover letter, CAPA survey, Web link on the Arthritis Society of Canada website, Web survey, and the 10 consumer summaries can be obtained from the authors. Correspondence assured respondents of the confidentiality of their data and stated that the survey had been approved by the Ottawa Hospital Research Ethics Board.

All survey items were provided in a consistent order for all participants; no randomization of questions was carried out. All participants saw the same questions; no adaptive questioning or question branching was required. Completion of items was not enforced and the options “not applicable,” “no opinion,” and “rather not say” were included for all items comprising preexisting scales. Respondents were not asked to review responses before submitting the survey. No incentives were provided for respondents to complete the survey.

### Data Collection and Statistical Analysis

The online survey compiled respondent data automatically into a Microsoft Excel file maintained by the Arthritis Society Web master and provided to the study researchers. Data collected were anonymous, organized by identification numbers created by the Arthritis Society. Access to data was limited to the principal investigators and one research assistant. No technical methods were used to prevent multiple entries from the same individual. The reviewing agency flagged the use of “cookies” (ie, small pieces of software code placed on the user’s machine from the survey server intended to track usage) as a potential ethical concern. The authors determined that the inclusion of cookies as a method of determining who had previously completed the survey adds little information over and above the use of the other acceptable methods (Web traffic logs or a specific question on the survey) and the use of cookies was not implemented for this study. As a result, the CAPA email survey provided a website link for respondents who previously completed the survey elsewhere, which advised against duplicate entries. Incomplete surveys were assessed manually by viewing Excel data files to determine whether any respondents had stopped short of completing the survey and simply submitted what had been completed. No statistical correction for nonrepresentative sampling was computed. Surveys were presented as a single webpage requiring 1 submission of data upon completion; therefore, it was not possible to determine rates of how many people had agreed to submit but had not submitted their final data. Nonresponder information was not available, making it impossible to compute view rates or participation rates for the Web administered survey. Time to complete the survey was not computed, as a time stamp was given at time of submission of the survey only.

Survey data were collected between August 1, 2005, and February 28, 2006. Closed-ended items were analyzed using frequencies and descriptive statistics using SPSS, version 16 (SPSS Inc, Chicago, IL, USA) and SAS, version 9.1 (SAS Institute Inc, Cary, NC, USA). Missing data were assessed on a question-by-question basis. Open-ended comments about how to improve the summaries were assembled into Microsoft Excel and examined for themes. In the next step, 3 coders (authors JB, AL, NS) reviewed all comments provided by respondents and coded them into 1 or more of 12 themes determined by an initial scan of the responses. Disagreements over how comments should be categorized into themes were resolved by consensus. We employed the Checklist for Reporting Results of Internet E-Surveys (CHERRIES) reporting guideline to inform our report of this study [37].

## Results

Over the 7-month period from August 2005 through February 2006 that the survey was available on the Arthritis Society website, 162 site visitors responded to the survey; the number of hits to the survey-relevant summaries during this time period was not collected. Of the invitations we sent to the CAPA members, we obtained 99 responses out of 395 successfully delivered emails (25%). After comparisons of demographic characteristics showed no important differences between the website and CAPA respondents, responses from the 2 groups were combined for a total of 261 responses.

Table 2 describes the background and summary-specific characteristics of the respondents. The majority (176/261 or 67%) of respondents were female, and 56% (147/261) were between the ages of 45 and 64 years of age. Most were well educated, with 77% (202/261) of respondents reporting at least some postsecondary education, and 35% (92/261) were employed full-time. Respondents were distributed across western (British Columbia, Alberta, Saskatchewan, Manitoba; 49/261 or 19%), central (Ontario, Quebec; 61/261 or 23%), and eastern Canada (New Brunswick, Nova Scotia, Prince Edward Island, Newfoundland; 115/261 or 44%) and included respondents from all 10 provinces, but none from the 3 northern territories. Approximately 7% (17/261) of respondents were not from Canada.

Respondents showed variable computer experience, but 48% (125/261) reported a moderate level of experience. A substantial majority (226/261 or 87%) reported that they themselves had some form of arthritis. Of the 10 different target summaries, 48% (124/261) of respondents chose a summary focused on rheumatoid arthritis, while another 41% (107/261) chose osteoarthritis. Nearly half (111/261 or 48%) reported spending less than 5 minutes reading the summaries. The most common ways in which the summaries were discovered were by simple exploration of the Arthritis Society of Canada website (60/261 or 23%), upon physician recommendation (55/261 or 21%), or through support groups like CAPA (44/261 or 17%), while the most common reasons for reading the summaries were for personal relevance (169/261 or 65%) or general interest (51/261 or 20%).

**Table 2:** Characteristics of respondents

Characteristic		% of Respondents n = 261
**Sex**		
	Female	67.4
	Male	17.6
	Missing	14.9
**Age (years)**		
	< 45	21.5
	45 to 54	26.4
	55 to 64	29.9
	65 and over	9.2
	Missing	13.0
**Education**		
	High school or less	9.6
	Some postsecondary	36.0
	Bachelor’s degree or higher	41.4
	Missing	13.0
**Employment**		
	Full-time	35.3
	Part-time	9.6
	Retired	18.4
	Disability leave	14.2
	Unemployed	4.2
	Other	2.7
	Missing	15.7
**Geographic location in Canada**		
	Western	18.8
	Central	23.4
	Eastern	44.1
	Outside Canada	6.5
	Missing	7.3
**General computer experience**		
	Low	15.7
	Moderate	47.9
	High	25.3
	Missing	11.1
**Arthritis experienced by**		
	Respondent	86.6
	Other	5.4
	Missing	8.1

**Information sought for**		
	Rheumatoid arthritis	47.5
	Osteoarthritis	41.0
	Other	11.5
**Time spent reading the summary (minutes)**		
	< 5	42.5
	5 to 10	34.1
	> 10	11.9
	Missing	11.5
**Summary discovered by**		
	Exploring arthritis.ca website	23.0
	Physician recommendation	21.1
	Support group recommendation	16.9
	Search engine	5.4
	Friend/family recommendation	3.8
	Other	16.1
	Missing	13.8
**Reason for reading the summary**		
	Personal relevance	64.8
	General interest	19.5
	Other	4.2
	Missing	11.5

Table 3 describes the number and percentage of the 261 respondents reviewing each consumer summary and self-reported time spent reviewing each summary. Of the 10 consumer summaries, 3 accounted for 54% (141/261) of respondents. A significant proportion of respondents spent less than 5 minutes reviewing each summary (summary-specific range of the percent of respondents who spent less than 5 minutes reviewing the summary, 25% to 70%).

**Table 3:** Number and percentage of respondents reviewing each consumer summary and self-reported time spent reviewing that summary page

Summary Title	Reviewed the Summary n (% of 261)	Reported Spending ≤ 5 Minutes Reviewing the Summary n (% of 261)	Reported Spending > 5 Minutes Reviewing the Summary n (% of 261)	Did Not Report Time Viewing the Summary n (% of 261)
Does exercise help osteoarthritis of the hip or knee?	52 (19.9%)	29 (55.8%)	20 (38.5%)	3 (5.8%)
Does glucosamine work for treating osteoarthritis?	45 (17.2%)	19 (42.2%)	24 (53.3%)	2 (4.4%)
Does methotrexate work to treat rheumatoid arthritis?	44 (16.9%)	11 (25.0%)	19 (43.2%)	14 (31.8%)
Does etanercept work to treat rheumatoid arthritis?	30 (11.5%)	11 (36.7%)	18 (60.0%)	1 (3.3%)
Does physical therapy work to treat ankylosing spondylitis?	20 (7.7%)	6 (30.0%)	10 (50.0%)	4 (20.0%)
Does folic acid decrease side effects in patients taking methotrexate for rheumatoid arthritis?	19 (7.3%)	8 (42.1%)	9 (47.4%)	2 (10.5%)
Does occupational therapy help people with rheumatoid arthritis?	17 (6.5%)	9 (52.9%)	6 (35.3%)	2 (11.8%)
Do antimalarials work to treat rheumatoid arthritis?	14 (5.4%)	7 (50.0%)	7 (50.0%)	0 (0.0%)
Do steroid injections work to treat tennis elbow?	10 (3.8%)	7 (70.0%)	2 (20.0%)	1 (10.0%)
Does ultrasound therapy work to treat osteoarthritis of the knee?	10 (3.8%)	4 (40.0%)	5 (50.0%)	1 (10.0%)
Total number (%) of respondents	261 (100%)	111 (42.5%)	120 (46.0%)	30 (11.5%)

Table 4 describes responses to the 14 user experience items. A number of items indicated favorable impressions by respondents, with a majority either strongly or moderately agreeing with statements that the summary used language that was appropriate (196/261 or 75%), provided expected information (178/261 or 68%), (didn’t) involve too much reading (172/261 or 66%), would be of use to many others (160/261 or 61%), was well laid out (159/261 or 61%), (wasn’t) hard to learn from (157/261 or 60%), (wasn’t) frustrating (154/261 or 59%)**,** was useful to me (153/261 or 59%) and (wasn’t) boring (149/261 or 57%). Somewhat fewer respondents agreed that they could find all the information they wanted (118/261 or 45% strongly or moderately agreed). Finally, several items suggested that many respondents felt unable to judge the credibility or reliability of the summaries. Relatively few agreed that the summary clearly provided information on the providers of the information (112/261 or 43%). Furthermore, large proportions of respondents selected “no opinion/don’t know” to whether the information is up-to-date (54/261 or 21%), the providers of the information are reliable (80/261 or 31%), and the information presented was the best available (68/261 or 26%).

**Table 4:** Percentage of respondents for items of user satisfaction (higher percentages indicate greater satisfaction)

Item of User Satisfaction	“Strongly Agree” to “Moderately Agree” % of 261	Less Than “Moderately Agree” % of 261	“No Opinion/ Don’t Know” % of 261	No Response % of 261
The summary uses appropriate language.	75.1	11.1	1.2	12.6
The summary provided the info I expected.	67.8	18.8	0.8	12.6
I had to read too much.^a^	65.9	20.3	0.8	13.0
This summary would be useful to many others.	61.3	24.1	1.5	13.0
The summary is well laid out.	60.9	23.8	1.9	13.4
Learning from this summary is hard.^a^	60.2	26.4	0.0	13.4
This summary is frustrating. ^a^	59.0	26.1	1.9	13.0
The information was useful to me.	58.6	28.4	0.4	12.6
This summary is boring. ^a^	57.1	28.7	0.8	13.4
The information is up-to-date.	51.7	14.9	20.7	12.6
I can easily find all the information I want.	45.2	39.9	1.9	13.0
The summary clearly presents who provides the information.	42.9	35.3	8.4	13.4
The providers of the information are reliable.	42.9	14.2	30.7	12.3
The information presented was the best available.	31.8	29.9	26.1	12.3

^a^ These items are reversed, that is, to the respondent, the questions appeared as written, but the scores reported in this table are reversed to ensure agreement percentages reflect a positive opinion of the summary for all items.

Table 5 describes responses to the items related to preparation for decision making. In general, the responses indicated that the summary would improve preparation for decision making, particularly to help to identify questions to ask the physician (157/261 or 60%). Relatively few agreed with the statement that the summary helps you know that your values affect your decisions (103/261 or 40%).

To probe in more detail issues around people’s impressions of the summaries, we asked respondents to provide us with feedback about how we might improve the summaries. Of the 261 respondents, 131 provided 1 or more comments. Table 6 presents the themes identified, the relative frequencies with which those themes were mentioned, and shows examples of comments relevant to each theme. The most commonly cited themes were to provide more detail about the treatment and options, risks and the research; to provide additional interactivity or functionality to the summaries; to make the language clearer or simpler; and to increase the use of pictures, graphs or colors. Several people specifically requested the summaries use point form and provide more bottom lines about the treatments, and several suggested that more detailed information about the credibility of the information should be provided.

**Table 5:** Percentage of respondents by satisfaction with preparation for decision making

The Summary Would:	“Quite a Bit” or “A Great Deal” % of 261	Less Than “Quite a Bit” % of 261	No Response % of 261
Help identify questions you want to ask	60.2	26.8	13.0
Help you think about how involved you want to be	55.2	32.2	12.6
Prepare you to communicate your opinion	54.4	32.6	13.0
Prepare you to make a better decision	51.3	36.0	12.6
Help you prepare for a follow-up visit	50.6	36.0	13.4
Help recognize that a decision needs to be made	47.1	40.2	12.6
Help make a follow-up visit run more smoothly	46.0	41.4	12.6
Help you think about pros and cons of the decision	45.2	39.9	14.9
Help you organize your own thoughts	44.8	42.5	12.6
Help you think about what is most important	43.3	44.1	12.6
Help you know that your values affect decisions	39.5	48.3	12.3

**Table 6:** Frequency of themes and examples of theme-relevant quotes of respondents’ impressions of the summaries (n = 131)

Theme	Frequency	Examples of Quotes
Need more information about treatment	40	*…length of time before effects are felt, how the meds can be taken...dosage of the meds (how often)...* [Respondent 175]
Need more information about risks, side effects, or contraindications	15	*The risk of treatment to patients should always be included. When I make a decision, I always want to know the risks involved.* [Respondent 140]
Need more information about research details	15	*Refer to other studies done or ones planned. More details on number of cases studied...* [Respondent 198]
Need more information about other treatment options	13	*It would be interesting to know how well treatments other than [X] perform for comparison.* [Respondent 85]
Need more information about the disease	8	*Try to explore the psychological issue with patients suffering [ankylosing spondylitis], such as depression, suicide, etc.* [Respondent 251]
Need additional interactivity, website functionality	20	*Maybe you could have a basic summary and have sections that expand if more detail is needed...* [Respondent 212]
Use pictures, graphs, or colors	12	*... though the statistics are interesting and indeed useful, perhaps presenting the information in a more visual manner...* [Respondent 182]
Make language clearer or simpler	12	*In an effort to use simple language, this summary was often vague and imprecise.* [Respondent 203]
Improve the format of the material	10	*A solid recommendation to do or not to do would help take away the uncertainty of decision making...* [Respondent 257]
Need more information about credibility	9	*Wasn’t real clear on the source of the information.* [Respondent 241]
Compliments	8	*I found [it] to be straightforward, easy to comprehend.* [Respondent 223]

## Discussion

The 15-year evolution of the CMSG consumer summaries has resulted in a successful standardized presentation format that enables brief but clear presentation of research evidence for a wide range of treatments and interventions for musculoskeletal disorders. Because these consumer summaries were carefully tested as paper-based tools but hadn’t been tested as Web-based tools, we decided to evaluate how they performed on the Web and what specific areas needed work in order to improve them as Web-based tools. Our survey showed that the tools were generally rated favorably and identified specific areas for improvement, which we discuss below.

### Amount of Detail

One key finding was that many respondents reported wanting additional information to be available from the summaries. Less than half of respondents (118/261 or 45%) agreed that they could easily find all the information they wanted. Open-ended comments also revealed that many respondents wanted more details about specific risks, about the types of studies comprising the research, and about the type of participants in the studies (presumably so that they could compare themselves to the study participants). Many respondents also wanted more information about the condition and its various treatment options.

We note that our sample of respondents was likely quite sophisticated in terms of its existing knowledge on arthritis-related issues. Over 77% (202/261) of respondents had some postsecondary education, and many came through recruitment from the Canadian Arthritis Patient Alliance group, members of which are likely to be actively engaged in issues of musculoskeletal disorders. This sample may, therefore, have been quite well informed already about issues around their illness (226/261 or 87% had personally experienced some arthritis condition) and, therefore, preferred relatively detailed information.

A key challenge when presenting any health information online is dealing with the variability in user needs. Nearly half (111/261 or 48%) spent less than 5 minutes reading the consumer summaries, and the clear majority (200/261 or 77%) reported spending 10 minutes or less reading them. The standardized format used here was designed primarily to yield clear, concise summaries of systematic reviews. Yet many users will wish to use these summaries as springboards for more detailed information searches. This need for more flexible, interactive information presentation was evident in our survey findings: 2 commonly cited themes were to increase use of interactivity and to include more in the way of graphs, charts, and other information-rich display formats. To facilitate such uses, it seems likely that rather than trying to create a one-size-fits-all solution that would be too detailed for some and not detailed enough for others, the challenge for developers is to provide a flexible, interactive approach that can allow users to tailor for themselves the amount and type of information they review [38]. This approach is becoming increasingly feasible on the Web, and suggests a clear avenue for future research.

We have begun this work in at least 3 ways. First, we are exploring the utility of providing links within these summaries to other, more detailed descriptions of the systematic reviews. Second, the consumer summaries are housed within the larger Arthritis Society website that includes a wealth of information on all aspects of musculoskeletal disorders; efforts to link from the summaries to this additional information may well improve areas identified in this survey. Third, we are currently conducting research on how to adapt more detailed decision support tools such as patient decision aids for use online, discussed in more detail below.

### Layout and Language

While the majority of respondents felt our consumer summaries incorporated clear language and were well laid out, some respondents did feel that the language or formatting could be improved. Within the CMSG, we have avoided evaluating the summaries using readability algorithms due to the number of technical, often complex terms at the heart of the reviews. Instead, our goal has been to use relatively few content words per sentence (ie, use lower lexical density) and a clear, logical progression from background information to the effects of treatment [29]. While this approach appears to be a qualified success, there may be opportunity for linking terms; such an approach can allow optional, more detailed information and definitions to be provided without adding length or clutter. We are currently exploring the use of such techniques in another study.

### Credibility of the Source

Many people were unable to judge the credibility or reliability of the summaries or did not know who the providers of the information were. This is a concern since assessment of the credibility of online information is a key component of evaluating health information [24]. Our summaries included a statement that the research is based on a Cochrane review, a reference to the review at the bottom of the summary, and a link to a website, About Cochrane, that describes the processes involved in writing a review. Clearly some respondents did not see or make use of this information; more investigation of how to make this information more salient to users will be important.

### Preparation for Decision Making

While user experience was generally positive, fewer than half of the respondents felt that the summary helped them recognize that a decision needs to be made, think about pros and cons, know how their values affect their decision, organize their own thoughts, or prepare to make a better decision. It is unsurprising that these summaries should not have all information necessary to prepare people for a decision since they are limited chiefly to providing information about the treatment options and the pros and cons of the treatments [39]. We are currently assessing whether patient decision aids, that is, decision support tools designed to help people make specific and deliberative choices among options, may be useful when presented online. While their effectiveness has been demonstrated in a variety of other presentation formats [20], it remains an open question how such tools can most effectively be employed via the medium of the Web and for what situations such tools may be most useful. These tools not only present information on the options and outcomes relevant to the person’s health status but often also include exercises to help patients explicate factors such as how they value the different options, preference for role in decision making, or choice predisposition. Patient decision aids can be much more detailed and, therefore, longer than consumer summaries. Use of these more detailed tools may be warranted in situations where a decision cannot be made on the basis of a consumer summary.

### Limitations

The response rate and makeup of our sample of respondents is one clear limitation of the present study. The Web subsample was collected over a period of 7 months from summaries that see hundreds of visitors per month. While we have no information on nonresponders, we have to assume that our relatively slow accrual rate suggests that we were only obtaining data from a small, select group of visitors to the site. The CAPA survey subgroup likely exhibits similar biases. Despite our best efforts, logistical limitations prevented us from obtaining a high response rate from the CAPA survey sample. The response rate (99/395 or 25%) suggests that we may have a very select sample of the CAPA group, which itself is likely quite different from the target population of all arthritis patients using online information. In total, we must assume that our sample is biased with respect to our overall target population. We have chosen to interpret our results not as a sample representative of all arthritis sufferers, but as one of a relatively sophisticated sample of well-educated patients. Future work will be needed to assess whether these findings generalize to the wider population of arthritis patients who use online information.

A second clear limitation of the current work is the lack of a control group against which to compare the survey findings. We chose the single-group design in order to evaluate the CMSG model for presenting consumer information online as it is currently being implemented on the Canadian Arthritis Society website, and we chose to use the information derived from it to inform future controlled studies. Our use of previously validated measures (eg, computer experience and satisfaction with preparation for decision making) gives us confidence in the constructs we have measured, but ongoing work using controlled designs will assess the extent to which the levels of these constructs can be improved upon using other approaches.

### Conclusions

The relationship between the Cochrane Musculoskeletal Group and the Arthritis Society has created an excellent opportunity for research producers to target those people who would benefit most from this research information. The current work focuses specifically on engaging in this knowledge translation process in an online environment and makes clear that while we are on the right track, there is more work to do in order to understand how best to communicate systematic review information online. We have begun this work and provided some initial recommendations about how consumer summaries should look. Feedback from this survey will help to reshape and improve on the current presentation format for consumer summaries used by the CMSG. Our results should also provide initial guidelines to other developers of patient information who wish to reach consumers via the Internet.

## Abbreviations

AHRQ: (US) Agency for Healthcare Research and Quality
CAPA: Canadian Arthritis Patient Alliance
CHERRIES: Checklist for Reporting Results of Internet E-Surveys
CSMG: Cochrane Musculoskeletal Group
NICE: (UK) National Institute for Health and Clinical Excellence
